# HIV-1 Env DNA prime plus gp120 and gp70-V1V2 boosts induce high level of V1V2-specific IgG and ADCC responses and low level of Env-specific IgA response: implication for improving RV144 vaccine regimen

**DOI:** 10.1038/emi.2017.90

**Published:** 2017-11-29

**Authors:** Qian Wang, Yanyan Dai, Zhiwu Sun, Xiaojie Su, Yufeng Yu, Chen Hua, Wei Xu, Shibo Jiang, Lu Lu

**Affiliations:** 1 Key Laboratory of Medical Molecular Virology of MOE/MOH, School of Basic Medical Sciences & Shanghai Public Health Clinical Center, Fudan University, Shanghai 200032, China; 2 Lindsley F. Kimball Research Institute, New York Blood Center, New York, NY 10065, USA

**Dear Editor,**

According to UNAIDS, >35 million people have died of AIDS, and about 36.7 million people were still living with HIV worldwide by the end of 2015. Therefore, it is essential to develop an efficacious vaccine to prevent HIV infection. However, even with numerous HIV vaccine clinical trials having been completed, no HIV vaccine has successfully emerged.^1, 2^ Notably, the RV144 HIV vaccine regimen, which comprises a recombinant viral vector vaccine ALVAC-HIV (vCP1521) prime and bivalent gp120 vaccine AIDSVAX B/E boost showed an efficacy of 31.2% reduction of HIV-1 infection in clinical trial.^2, 3^ Inspired by these promising results, a large-scale HIV vaccine trial (HVTN 702), based on the RV144 vaccine regimen tested in clinical trials in Thailand, will be launched in South Africa.^4^

Studies on the RV144 vaccine regimen revealed that this vaccine does not induce effective neutralizing antibody responses, but it does elicit antibody-dependent cell cytotoxicity (ADCC) effect, which may play a key role in preventing HIV infection. It was demonstrated that the protection against HIV-1 infection was directly correlated with the level of IgG specific for the HIV-1 gp120 V1V2 region (V1V2-specific IgG, V1V2-IgG) and inversely with the level of IgA specific for HIV-1 envelope protein (Env-specific IgA, Env-IgA) in vaccine recipients.^5^ These results suggest that V1V2-IgG, Env-IgA and ADCC^6^ may be important parameters associated with the protective effect induced by RV144 vaccine and that a more effective and safe HIV vaccine regimen could be designed and developed on the basis of these parameters.

We previously designed an effective and safe subunit vaccine candidate against Middle East respiratory syndrome coronavirus by using an immunofocusing strategy, that is, identifying the shortest fragment in Middle East respiratory syndrome coronavirus spike protein containing the critical epitopes that induce protective immune responses, while eliminating the fragment(s) with epitopes that may elicit non-protective or harmful immune responses.^7^ Here we applied this immunofocusing strategy to design a DNA prime/protein boost vaccine regimen. To induce immune response focusing on V1V2 region in gp120, additional boosts with gp70-V1V2 that contains the V1V2 region was applied after the primes with HIV-1 Env-coding DNA (pEnv) and boosts with HIV-1 gp120 (Figure 1A).

Two groups of New Zealand rabbits (*n*=3, 6–8 weeks of age, ~2 kg) were intradermally immunized with the following vaccine regimens, respectively (Figure 1A): (1) Prime three times with a plasmid encoding HIV-1 JRFL Env with gp41 cytoplasmic domain truncated (pEnv) and boost twice with gp120, as well as boost twice with gp70-V1V2 of the HIV-1 JRFL (pEnv+gp120+gp70-V1V2). Both gp120 and gp70-V1V2, which were expressed in 293 T cells, were obtained from Jiangsu Haiyuan Company, Taizhou, China. (2) Prime three times with pEnv and boost four times with gp120 (pEnv+gp120). The gp70-V1V2 is mainly composed of V1V2 region (residues 157–196, according to HXB2 sequence number) in gp120 and sequences at the two ends that can form a scaffold structure to stabilize its conformation, which has been clinically used to detect antibodies against V1V2.^5, 8^ Four additional groups of New Zealand rabbits were intradermally immunized with the following vaccine regimens, respectively, as controls: (1) prime three times with pEnv and boost four times with gp70-V1V2 (pEnv+gp70-V1V2); (2) prime once and boost four times with gp120 (gp120); (3) prime once and boost four times with gp70-V1V2 (gp70-V1V2), and (4) prime once and boost four times with phosphate-buffered saline (PBS) (Figure 1A).

To evaluate the efficacy of the above vaccine regimens to induce V1V2-IgG, rabbit sera from different immunization groups were collected 10 days after the last boost for testing the titers of the antisera to bind gp70-V1V2. As shown in Figure 1B, the gp70-V1V2 and pEnv+gp70-V1V2 groups exhibited the highest titer of antibody responses, followed by the pEnv+gp120+gp70-V1V2 group and then the pEnv+gp120 and gp120 groups. Next, we purified the total IgG in sera from different groups and tested their binding capacity to gp70-V1V2. The results show that at the same concentration (10 μg/mL), the gp70-V1V2-binding activity of the total IgG from gp70-V1V2 and pEnv+gp70-V1V2 groups is the highest, followed by that from the pEnv+gp120+gp70-V1V2 group and then those from the pEnv+gp120 and gp120 groups (Figure 1C), which is consistent with that of the antisera.

As antibodies binding to V1V2 region of the HIV-1 Env play a key role in mediating ADCC effect, V1V2-specific IgG (V1V2-IgG) was purified with NHS-activated Sepharose 4 Fast Flow beads (GE Healthcare, Stockholm, Sweden) coated with gp70-V1V2 as previously described.^9^ HIV-1 JRFL-infected CEM.NKR-CCR5 cells (HIV-1-infected cells) were used as target cells for detection of V1V2-IgG and Env-IgA binding, respectively, by flow cytometry.^9^ As shown in Figure 1D, at the same concentration, V1V2-IgG from the pEnv+gp120+gp70-V1V2 group exhibited the highest level of binding to the HIV-1-infected cells, followed by that from the pEnv+gp120 group and then that from the pEnv+gp70-V1V2 group. V1V2-IgG from the gp120 group and that from the gp70-V1V2 group showed moderate, or marginal, level of binding to HIV-1-infected cells.

It was reported that the key binding site of the neutralizing mAb PG9 is in the V1/V2 region in the HIV-1 Env on HIV-infected cells.^10^ Therefore, we used PG9 as the known V1V2 antibody in a competition assay to compare its V1V2-binding activity with that of V1V2-IgG induced by those vaccine regimens. In brief, V1V2-IgG at a concentration of 0, 10 and 20 μg/mL, respectively, was preincubated with the HIV-1-infected cells H9IIIB at 4 °C for 60 min, before addition of PG9 at 10 μg/mL, followed by detection of the PG9-binding cells using the fluorescence-labeled secondary antibody. As shown in Supplementary Figure S1, V1V2-IgG purified from the sera of pEnv+gp120+gp70-V1V2 group exhibited the highest inhibition on PG9 binding to the HIV-1-infected cells, followed by that from the pEnv+gp120 and pEnv+gp70-V1V2 groups and then from the gp120 and gp70-V1V2 groups. This result is consistent with that from the V1V2-IgG-binding experiment (Figure 1D), confirming that the pEnv+gp120+gp70-V1V2 vaccine regimen can induce high level of IgG antibodies binding to V1V2 region of the HIV-1 Env.

The above results suggest that the additional gp70-V1V2 boost after the pEnv prime/gp120 boost is important to enhance V1V2-IgG response. Meanwhile, the result indicates that the gp120 boost is also essential to induce functional V1V2-IgG that can bind to the native conformational epitopes in the V1V2 region of Env on the surface of HIV-1-infected cells since V1V2-IgG from the pEnv+gp70-V1V2 group is less effective in binding to the HIV-1-infected cells than that from pEnv+gp120+gp70-V1V2. Prime and boosts with gp70-V1V2 only could not induce functional V1V2-IgG response (Figure 1D). It is possible that the conformation of the V1V2 region in gp120 may be more like to the native conformation of V1V2 region in the Env of virions or HIV-1-infected cells than that of the V1V2 region in gp70-V1V2. Therefore, the V1V2 in gp120 may bind to intermediate B-cell receptor more easily than that in gp70-V1V2. However, the V1V2 region in gp70-V1V2 may be more accessible to B-cell receptor trained by the V1V2 region in gp120. This may explain why the pEnv prime/gp120 boost/gp70-V1V2 boost vaccine regimen elicits more effective V1V2-IgG response than the pEnv prime/gp70-V1V2 boost and gp70-V1V2 prime/gp70-V1V2 boost vaccine regimens. These results suggest that gp70-V1V2 and pEnv+gp70-V1V2 immune regimens induce higher levels of antibodies binding to gp70-V1V2 protein, while pEnv+gp70-V1V2 immune regimen elicits higher level of antibodies binding to the native conformation of V1V2 region in the HIV-1 Env. Therefore, the pEnv+gp120+gp70-V1V2 regimen should be selected for further study.

At the same time, we also used flow cytometry to test the sera of each group for the binding of Env-IgA to HIV-1-infected cells. As shown in Figure 1E, Env-IgA from the gp120 prime/gp120 boost group and that from the gp70-V1V2 prime/gp70-V1V2 boost group displayed the highest and lowest levels of binding to HIV-1-infected cells, respectively. This result suggests that the epitopes that elicit Env-IgA are located in gp120 outside the V1V2 region. Interestingly, the Env-IgA from the pEnv prime/gp120 boost/gp70-V1V2 boost group exhibited significantly lower level of binding to HIV-1-infected cells than that from the pEnv prime/gp120 boost group (*P*<0.05), suggesting that gp70-V1V2 in the vaccine regimen may have a suppressive effect on Env- or gp120-induced Env-IgA response.

Subsequently, we tested ADCC effect on HIV-1-infected cells mediated by natural killer (NK) cells and V1V2-IgG from the pEnv+gp120+gp70-V1V2 group, the pEnv+gp120 group, and PBS control group, respectively Env+gp120, and PBS control groups, respectively. After binding to the V1V2 region, V1V2-IgG, via its Fc fragment, then binds and activates the Fc receptor FcγRIIIa (CD16) on NK cells, which results in killing HIV-1-infected cells. Therefore, we first assessed the ability of HIV-1-infected cell binding V1V2-IgG to activate FcγRIIIa with an FcγRIIIa stimulation assay, using an ADCC Reporter Bioassay Kit (Promega, Madison, WI, USA). HIV-1-infected cells were incubated with V1V2-IgG before co-culture with Jurkat NFAT-lucFcγRIIIa cells expressing an NFAT-luciferase reporter, which can be activated by FcγRIIIa stimulation.^9^ As shown in Figure 1F, the HIV-1-infected cell-binding V1V2-IgG from the pEnv+gp120 group could effectively activate FcγRIIIa on Jurkat NFAT-luc+FcγRIIIa cells, compared with that from the PBS group, while the V1V2-IgG from the pEnv+gp120+gp70-V1V2 group exhibited a significantly higher level of FcγRIIIa activation than that from the pEnv+gp120 group (*P*<0.05). Using the CytoTox-ONE Homogeneous Membrane Integrity Assay (Promega), we then further tested the activity of NK cells via ADCC effect to kill the V1V2-IgG-bound HIV-infected cells.^11^ HIV-1-infected cells were incubated with V1V2-IgG before co-culture with rabbit peripheral blood mononuclear cells containing NK cells. The activity of lactate dehydrogenase released from the HIV-1-infected cells killed by NK cells was measured using the same assay.^11^ Consistent with the result from the FcγRIIIa stimulation assay (Figure 1F), the ADCC mediated by NK cells and the V1V2-IgG from the pEnv+gp120+gp70-V1V2 group exhibited significantly stronger killing of the HIV-1-infected cells than that from the pEnv+gp120 group (*P*<0.05; Figure 1G). These results suggest that the additional gp70-V1V2 boost after the pEnv prime/gp120 boost is essential to enhance V1V2-IgG-mediated ADCC. Meanwhile, we performed an experiment to detect V1V2-IgG-mediated ADCC effect on target cells infected by different HIV-1 strains, including the laboratory-adapted strain Bal and the clinical strain 11244 (HIV-1 KNH1088). As shown in Figure 1H and 1I, V1V2-IgG induced by pEnv+gp120+gp70-V1V2 vaccine regimen mediated similar high level of ADCC effect to kill the cells infected by the HIV-1 Bal or 11244 strain, respectively, suggesting that V1V2-IgG could mediate a broad spectrum of ADCC effect against cells infected by laboratory-adapted or clinically isolated HIV-1 stains.

To mimic the RV144 vaccine regimen, we have designed a DNA prime and protein boost vaccine regimen. However, we also applied the immunofocusing strategy by adding final boosts with gp70-V1V2, consisting of V1V2 region in gp120, after pEnv prime/gp120 boosts, in anticipation of enhancing V1V2-IgG and ADCC responses, as well as reducing Env-IgA response. Indeed, we found that the levels of V1V2-IgG response and ADCC effect in the pEnv+gp120+gp70-V1V2 group were significantly higher compared with pEnv+gp120, while the level of Env-IgA response in the pEnv+gp120+gp70-V1V2 group was significantly lower than that in the pEnv+gp120 group. The immunofocusing strategy may promote the production of V1V2-IgG that can recognize and bind to V1V2 region with native conformation in the HIV-1 Env on HIV-1-infected cells, thus mediating the ADCC effect to kill these infected cells.These results suggest that the immunofocusing strategy is very helpful for designing a vaccine regimen by maintaining the fragment(s) in the immunogen containing the critical epitope(s) that elicit(s) protective immune responses, while excluding the fragment(s) containing the epitope(s) that induce(s) non-protective or harmful immune response but without sacrificing domain integrity and stability. Therefore, we prospect that this immunofocusing strategy may also be used to improve the RV144 HIV vaccine regimen, that is, addition of a final boost with gp70-V1V2 or similar recombinant protein containing V1V2 region in gp120, and that this improved RV144 HIV vaccine regimen may induce enhanced protective immune responses and reduced harmful immune response in the volunteers participating in the South Africa HVTN 702 clinical trials, which is based on the RV144 vaccine regimen.^4^

## Figures and Tables

**Figure 1:**
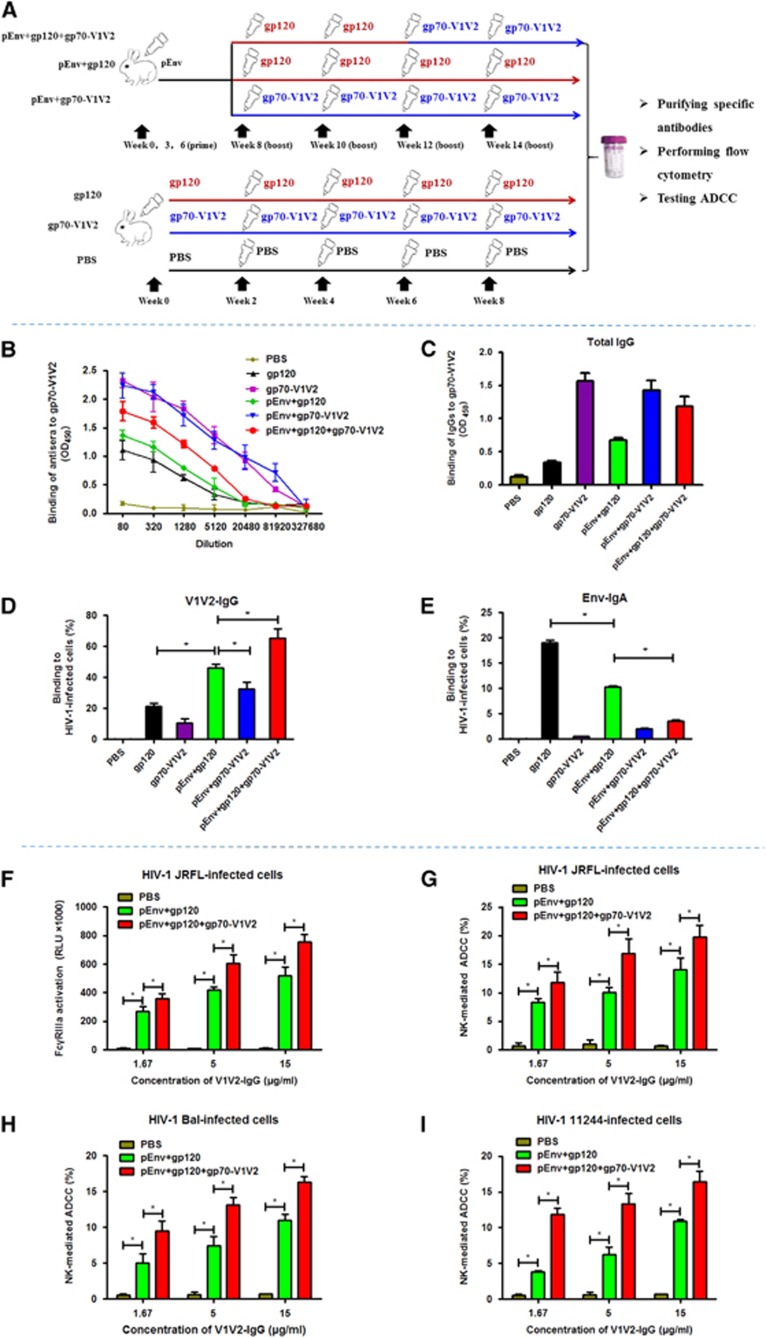
Immune responses induced by the pEnv prime/gp120 boost/gp70-V1V2 boost vaccine regimen. (**A**) Immunization regimens and protocol. We used 200 μg of pEnv for one rabbit at each prime, and 100 μg of gp120 or 200 μg of gp70-V1V2 for one rabbit at each boost (or prime). The Freund’s adjuvant (Sigma) was used for boost with a protein. (**B**) Binding of immunizing sera to gp70-V1V2. (**C**) Binding of total IgG to gp70-V1V2. (**D**) Binding of V1V2-IgG to HIV-1-infected cells. (**E**) Binding of Env-IgA to HIV-1-infected cells. (**F1**) V1V2-IgGbound to HIV-1 JRFL-infected cells for mediating activation of FcγRIIIa, the Fc receptor on Jurkat cells. (**G**) Killing of HIV-1 JRFL-infected cells mediated by V1V2-IgG and NK cells in rabbit peripheral blood mononuclear cells (PBMCs) via antibody-dependent cell cytotoxicity (ADCC) effect. (**H**) Killing of HIV-1 Bal-infected cells mediated by V1V2-IgG and natural killer (NK) cells in rabbit PBMCs via ADCC effect. (**I**) Killing of HIV-1 11244-infected cells mediated by V1V2-IgG and NK cells in rabbit PBMCs via ADCC effect.The samples were tested in triplicate, and the experiment was repeated twice. The data are presented as mean±s.d. *P<0.05. optical density, OD; phosphate-buffered saline, PBS.
