# Supplementary figures and images for: HIV-1 Env DNA prime plus gp120 and gp70-V1V2 boosts induce high level of V1V2-specific IgG and ADCC responses and low level of Env-specific IgA response: implication for improving RV144 vaccine regimen
Source: Emerg Microbes Infect. 2017 Nov 29;6(11):e102–. doi: 10.1038/emi.2017.90 (PMC5717091; doi:10.1038/emi.2017.90)

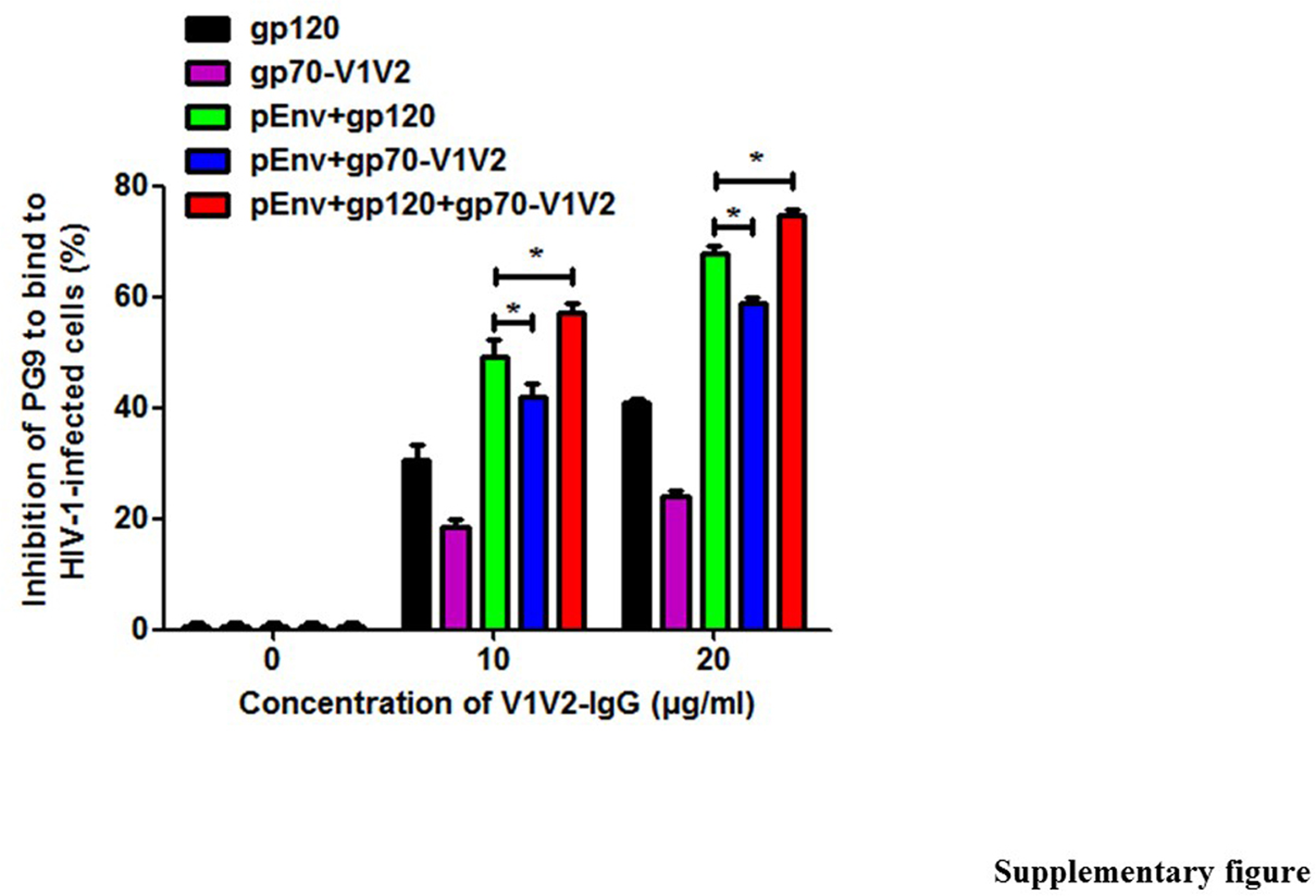

Supplement: Supplementary Figure S1 [file emi201790x1.tif]
